# Hybrid SES–MEW Scaffold Strategies: A Narrative Review of Multi-Scale Fiber Architectures for Soft and Hard Tissue Engineering

**DOI:** 10.3390/ph19050683

**Published:** 2026-04-27

**Authors:** Elisa Capuana, Valerio Brucato, Vincenzo La Carrubba

**Affiliations:** Department of Engineering, University of Palermo, Viale Delle Scienze, Ed. 8, 90128 Palermo, Italy; valerio.brucato@unipa.it (V.B.); vincenzo.lacarrubba@unipa.it (V.L.C.)

**Keywords:** solution electrospinning, melt electrowriting, hybrid scaffolds, tissue engineering, extracellular matrix mimicry, multi-scale fiber architecture, scaffold fabrication

## Abstract

Solution electrospinning (SES) and melt electrowriting (MEW) are complementary fiber-based fabrication platforms extensively investigated in tissue engineering. SES generates fibers typically ranging from the nanometer to the low-micrometer scale, producing fibrous networks that mimic the native extracellular matrix (ECM) and support key cellular functions. MEW, by contrast, operates solvent-free and enables precise, layer-by-layer deposition of microfibers with well-controlled geometry, conferring the mechanical integrity and open-pore architecture that SES constructs inherently lack. Despite growing interest, the body of peer-reviewed literature reporting original hybrid SES–MEW fabrication and biological outcome data remains limited, with no comprehensive cross-tissue synthesis available to date. This narrative review examines the current state of SES–MEW hybrid strategies across five tissue engineering targets selected for their clinical relevance: skin, vascular grafts, bone, cartilage, cardiac valves, and skeletal muscle. For each application, the architectural rationale, the fabrication approach, and the in vitro and in vivo biological outcomes are discussed in an integrated manner, with attention to how the spatial organization of nano- and microfibers translates into tissue-specific functional responses. A comparative analysis across tissue types highlights both the versatility of hybrid constructs and their persistent limitations, including suture retention values that remain below clinically accepted thresholds in vascular applications, incomplete cellular infiltration through dense nanofibrous layers, and the absence of validated, reproducible scale-up protocols compatible with clinical-grade manufacturing. The review concludes by identifying the most critical open questions in the field, encompassing process standardization, regulatory classification, and the emerging role of machine learning in closed-loop MEW process optimization. This work aims to provide an evidence-based perspective on the current state of hybrid SES–MEW scaffold engineering and the key translational gaps limiting clinical application.

## 1. Introduction

Engineering functional biological substitutes for damaged or degenerate tissues remains a major challenge in regenerative medicine, despite decades of advances in biomaterials, scaffold fabrication, and cell biology. At the heart of this challenge lies a fundamental tension: native tissues are hierarchical structures spanning multiple length scales, from collagen fibrils to whole-organ geometry [1,2].

Among the available scaffold manufacturing approaches, solution electrospinning (SES) has emerged as one of the most effective techniques for fabricating micro- and nanofibers characterized by high porosity, large surface area, and structural mimicry of the extracellular matrix (ECM) [3]. These features directly promote cell adhesion, proliferation, migration, and phenotypic maintenance [4,5]. The ability of SES to produce fibers ranging from nanometers to micrometers in diameter, and to tune composition, orientation, and porosity over a broad parameter space, has made it applicable to virtually every major tissue target [6,7].

As the field matured, the intrinsic limitations of SES became increasingly evident and difficult to overcome. The dense and compact packing of electrospun fibers produces pore sizes that are systematically below the threshold required for efficient cellular ingress. As demonstrated by Liu et al., collector rotation can reduce mean pore diameter by as much as 65% relative to unaligned controls, yielding values consistently below the threshold for meaningful cellular infiltration, an effect particularly pronounced in aligned fiber configurations [8,9]. The functional consequences of this bottleneck have been confirmed in mechanistic studies showing that cells seeded onto aligned PCL meshes under static culture conditions infiltrate only the outermost scaffold layers even after ten weeks of culture [10,11,12]. Compounding this limitation, the mechanical performance of purely electrospun constructs is often insufficient for load-bearing or cyclically stressed environments: the mismatch between the nonlinear, anisotropic mechanical behavior of native tissues and the mechanical response of electrospun sheets represents a barrier that has been quantified for cardiac [13], vascular [14], and musculoskeletal applications [15].

It is in this context that melt electrowriting (MEW) has emerged as a conceptually distinct and technically complementary fabrication platform. MEW is a solvent-free additive manufacturing technique that combines electrostatic jet stabilization with melt extrusion, enabling layer-by-layer deposition of thermoplastic microfibers with higher spatial precision than conventional fused deposition modeling [16]. Shahverdi et al. demonstrated that MEW-fabricated PCL and PLA/PCL scaffolds exhibit well-ordered pore architectures and cytocompatibility in vitro, though validation with primary cells under physiologically relevant conditions remains necessary [17]. A particularly important advantage of MEW over SES is the complete absence of organic solvents, which eliminates cytotoxicity and residual solvent concerns relevant for implantable constructs [18]. Włodarczyk-Biegun and del Campo further showed that MEW scaffolds span tensile moduli from 5.6 to 360 kPa depending on fiber architecture, demonstrating a degree of mechanical programmability inaccessible to SES [19].

These complementary capabilities have driven efforts toward hybrid multi-scale scaffolds, in which SES provides biomimetic nanoscale ECM cues and MEW supplies ordered microscale architecture governing mechanical behavior and spatial organization [20,21,22]. Despite this progress, a cross-tissue synthesis of hybrid SES–MEW outcomes remains lacking. Existing work has tended either to address the two techniques in isolation or to report tissue-specific results without systematic comparison across anatomical domains. Recent attempts to print MEW scaffolds on complex geometries have revealed difficulties in controlling electric fields and print speed, restricting fabrication largely to flat or cylindrical forms and limiting anatomical design freedom [23,24,25]. These geometric constraints are compounded by a limited polymer library, historically dominated by polycaprolactone, although expansion toward composites and biologically derived materials has begun. In addition, standardized fabrication protocols across laboratories remain absent [17,26].

The present narrative review addresses this gap directly. The literature search was conducted across PubMed, Scopus, and Web of Science using the terms “melt electrowriting”, “solution electrospinning”, “hybrid scaffold”, and combinations thereof with individual tissue targets, without date restriction. Studies were included if they reported original fabrication data, scaffold characterization, and at least one biological outcome (in vitro or in vivo); reviews, conference abstracts, and studies reporting MEW or SES in isolation without hybrid integration data were excluded. For each tissue domain, between four and twelve primary studies met these criteria and form the evidential basis of the tissue-specific sections. Five tissue domains were selected on the basis of clinical relevance, the availability of published hybrid SES–MEW data, and the diversity of mechanical and biological design requirements they collectively represent: skin, vascular grafts, bone, cartilage and cardiac valves, and skeletal muscle. The review then draws a comparative analysis across tissue types to identify both the shared structural principles and the tissue-specific design challenges that define the frontier of this emerging field. It concludes with an assessment of the translational barriers that remain, including process standardization, regulatory classification, and the integration of machine learning for closed-loop MEW process optimization, an approach in which real-time sensor feedback is used to iteratively correct fiber deposition parameters and reduce inter-run variability. The aim is to provide an evidence-based map of where the field stands and what must be resolved before clinical applicability can be realistically pursued.

## 2. Technical Principles of the SES–MEW Combination

Solution electrospinning and melt electrowriting operate on fundamentally different physical principles, process distinct material phases, and generate fibers in non-overlapping dimensional ranges. In SES, a polymer dissolved in an organic solvent is loaded into a syringe and subjected to a high-voltage electrostatic field, typically ranging between 10 and 30 kV, applied between the needle tip and a grounded collector positioned at a distance of 10 to 25 cm [27,28]. When the electrostatic force overcomes the surface tension of the droplet at the needle tip, a Taylor cone forms and a charged polymer jet is ejected. During flight, the jet undergoes whipping instability and solvent evaporation, yielding solid fibers with diameters from 100 nm to approximately 10 µm [29]. The process depends on interdependent parameters including solution rheology, solvent volatility, applied voltage, tip-to-collector distance, and flow rate [30,31]. The resulting membrane is a nonwoven mat of randomly or directionally oriented fibers, characterized by high porosity and a large surface-area-to-volume ratio. However, pore size scales inversely with fiber diameter, making small-fiber SES scaffolds inherently prone to limited inter-fiber pore dimensions regardless of overall porosity [32].

**Figure 1:**
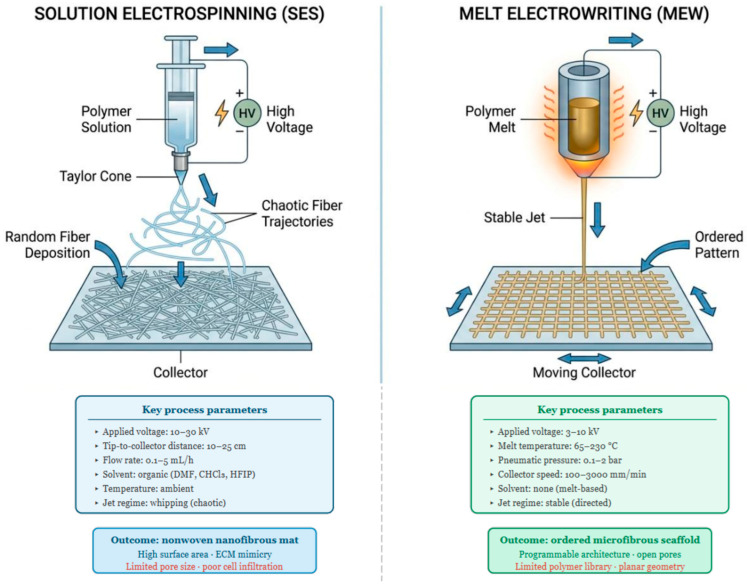
Schematic comparison of solution electrospinning (SES) and melt electrowriting (MEW) process principles. SES operates at ambient temperature by applying a high voltage (10–30 kV) to a polymer solution, inducing Taylor cone formation and whipping jet instability that drives solvent evaporation and fiber attenuation to the nanometric–micrometric range (Ø100 nm–5 µm). MEW operates solvent-free by extruding a polymer melt through a heated nozzle (65–230 °C) under lower voltage (3–10 kV) and controlled pneumatic pressure, stabilizing the jet into a directed filament deposited layer-by-layer onto a programmable collector (Ø3–100 µm). Key process parameters, fiber dimensional ranges, and principal advantages and limitations of each technique are indicated. SES: solution electrospinning; MEW: melt electrowriting; DMF: dimethylformamide; CHCl_3_: chloroform; HFIP: hexafluoroisopropanol.

In MEW, rather than dissolving the polymer, the material is heated above its melting point, typically between 65 and 230 °C, depending on the polymer type, and extrudes the melt through a heated nozzle under controlled pneumatic pressure, while simultaneously applying a voltage of 3 to 10 kV between the nozzle and the collector [33]. Operating above the critical translation speed, the electrostatic field stabilizes the jet into a straight, directed filament, enabling precise layer-by-layer deposition along a pre-programmed path [34]. As demonstrated by Xie et al. using an orthogonal experimental design across six parameters (melt temperature, collector speed, tip-to-collector distance, flow rate, voltage, and nozzle gauge), melt flow rate and collector speed are among the most influential parameters governing fiber diameter in MEW, with achievable diameters ranging from approximately 3 to 100 µm [35]. Parameter hierarchy, however, shifts with polymer type and system configuration, as confirmed by independent orthogonal analyses [36]. Böhm et al. further showed that for medical-grade poly(ε-caprolactone) (PCL), stable printing can be sustained for 25 days at 75 °C without significant drift in fiber diameter. When the collector speed is fixed to a value at least 10% above the jet speed, fiber diameter averages 13.5 ± 1.0 µm across the entire printing period; stability is not maintained at 85 or 95 °C, where progressive thermal degradation leads to diameter drift and fiber fusion at increased layer heights [37] (Figure 1).

The complementarity between the two techniques is what makes their integration architecturally compelling. SES produces sub-micrometric fibers that replicate the nanoscale topography of the ECM, offering high surface area for cell attachment, protein adsorption, and paracrine signal retention, but the resulting membranes systematically restrict cellular infiltration due to insufficient pore size. MEW, conversely, yields open-pore, geometrically defined microstructures with broadly tunable mechanical properties, but lacks the nanotopographic cues required for normal cell adhesion and phenotypic expression [38]. A comparative overview of the principal process parameters, structural outcomes, and translational attributes of SES, MEW, and their hybrid combination is provided in Table 1.

**Figure 2:**
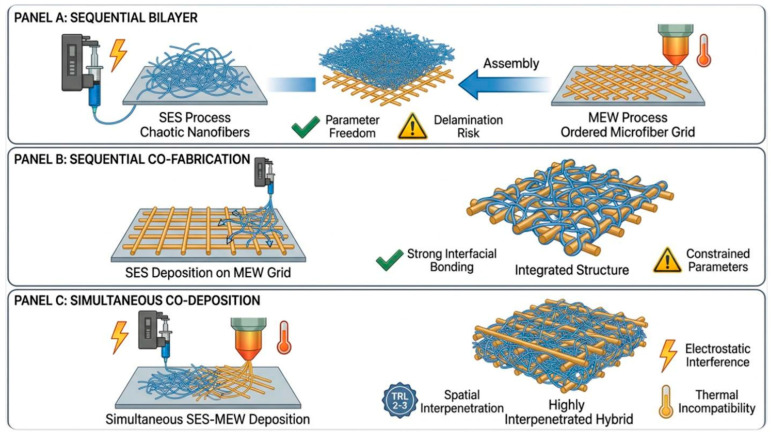
Schematic representation of the three fabrication strategies for hybrid SES–MEW scaffold integration. (**A**) Sequential bilayer: SES and MEW components are fabricated independently under fully optimized conditions and subsequently assembled into a layered construct; cohesion relies on mechanical interlocking of electrospun nanofibers into MEW pore interstices, supplemented by thermal annealing where applicable, with interfacial delamination under cyclic loading representing the principal failure mode. (**B**) Sequential co-fabrication: the MEW scaffold serves as a substrate onto which SES nanofibers are directly deposited following surface activation (e.g., oxygen plasma treatment), promoting physical entanglement and electrostatic adhesion of nanofibers into the MEW pore interstices and producing a nano–micro interdigitated interface with superior resistance to delamination relative to method A. (**C**) Simultaneous co-deposition: SES and MEW nozzles operate concurrently on a shared collector, enabling true spatial interpenetration of nano- and microfibers within a single scaffold layer; both populations are deposited while warm and charge-bearing, creating conditions for localized viscoelastic welding at nano–micro contact points and eliminating the discrete interfacial boundary inherent to methods A and B. This approach remains at an early technology readiness level (TRL 2–3) due to unresolved electrostatic field interference, orthogonal parameter conflicts, and thermal incompatibility near the collector surface. Representative bibliographic examples and principal trade-offs are indicated for each strategy. SES: solution electrospinning; MEW: melt electrowriting; TRL: technology readiness level.

The fabrication strategies for combining SES and MEW fall into three non-mutually exclusive categories, each with distinct manufacturing logic and specific trade-offs, as schematically illustrated in Figure 2. In the sequential bilayer approach, which is the most widely adopted, the two components are fabricated independently and then assembled, allowing each to be fully optimized in isolation before integration [39]. In this configuration, cohesion between layers is achieved primarily through mechanical interlocking: electrospun nanofibers penetrate the open-pore MEW microstructure under the applied pressure of stacking or compression bonding, generating a fibrous anchor zone at the interface [40]. Thermal annealing near the polymer glass transition temperature can supplement this with localized fiber fusion, though it risks altering fiber morphology and drug release kinetics if bioactive agents are incorporated [41]. In the sequential co-fabrication approach, one technique is applied directly onto the substrate produced by the other, achieving stronger interfacial bonding at the cost of constrained process parameters for the deposited component. In this approach, the MEW substrate is typically subjected to surface activation, such as oxygen plasma treatment (50–100 W, 60–90 s), prior to SES deposition [42]. This increases surface energy and promotes physical entanglement and electrostatic adhesion of freshly deposited nanofibers into the MEW pore interstices, producing a nano–micro interdigitated interface rather than a discrete planar boundary. A third and more technically demanding strategy involves truly simultaneous co-deposition from adjacent SES and MEW nozzles onto the same collector, enabling spatial interpenetration of nano- and microfibers within the same scaffold layer. This approach remains at TRL 2–3, facing unresolved charge interference between the two jets and the need to coordinate orthogonal process parameters within a shared electrostatic field [43]. In this configuration, both fiber populations are deposited while still warm and carrying residual charge, creating conditions for localized viscoelastic welding at nano–micro contact points and enabling structural continuity across the scaffold volume rather than at a defined interface [37]. In hybrid bilayer constructs, interfacial integrity has been identified as a critical determinant of both mechanical performance and in vivo patency, with delamination at the layer boundary representing a documented failure mode in sequentially assembled multi-material scaffolds under physiological loading [40,44]. Quantitative characterization of interfacial bond strength through peel testing, lap shear, or interfacial toughness measurements has not been reported for any SES–MEW hybrid construct reviewed here and represents a critical experimental gap; its absence precludes reliable prediction of delamination risk under physiological cyclic loading and constitutes a documentation requirement for implantable multi-layer constructs under MDR Annex [45,46].

## 3. Tissue-Specific Applications: A Critical Cross-Tissue Analysis

Examining SES–MEW hybrid scaffolds across multiple tissue targets simultaneously allows identification of whether their architectural logic follows tissue-specific rules or reflects shared structural principles. A cross-tissue reading of the available evidence reveals a more nuanced picture: while the nano–micro complementarity of SES and MEW is invoked across all applications, the way in which this complementarity is operationalized, and the biological outcomes it produces, differ substantially depending on the mechanical, cellular, and vascular demands of the target tissue. This section examines five tissue domains in sequence, with the explicit aim of distinguishing genuine advances from results that, while promising, remain insufficiently validated for translational purposes.

### 3.1. Skin

The skin represents the most structurally demanding target for bilayer scaffold design because of the precision with which its native stratified architecture must be recapitulated to achieve functional organ-level behavior. Central to this architecture is the dermoepidermal junction (DEJ), a specialized interface that provides mechanical interlock between layers, establishes stem cell niches for epidermal progenitors, and maintains a selective cellular boundary between dermis and epidermis [47]. Native dermal stiffness gradients (25–36 kPa at the DEJ) regulate basal keratinocyte fate via integrin-mediated adhesion [48]. Replicating this structural and mechanosensory complexity in a synthetic construct remains an unsolved problem that neither SES-only nor MEW-only scaffolds have satisfactorily addressed [49].

The most conceptually rigorous application of the SES–MEW combination to this problem is the bilayer scaffold reported by Girard et al. [50], comprising an upper SES layer of PCL nanofibers (282 ± 103 nm diameter; membrane thickness 13 ± 2 µm; pore size below 2 µm) positioned at the dermoepidermal interface to prevent keratinocyte infiltration while permitting nutrient and ECM exchange, and a lower MEW layer of straight or wavy PCL microfibers providing the open-pore dermal compartment for fibroblast seeding. The functional consequences are notable: keratinocytes can be seeded from day 1, bypassing the dermal pre-maturation period of 14 to 28 days required by collagen-based full-thickness skin equivalents under standard protocols, and elastin deposition is detectable at day 18, a timeline not previously reported in scaffold-based skin models [51]. The SES membrane therefore functions as a biomimetic surrogate for the DEJ basement membrane rather than a simple physical barrier, constraining cell compartmentalization in a way that accelerates ECM neosynthesis throughout the dermal volume.

**Figure 3:**
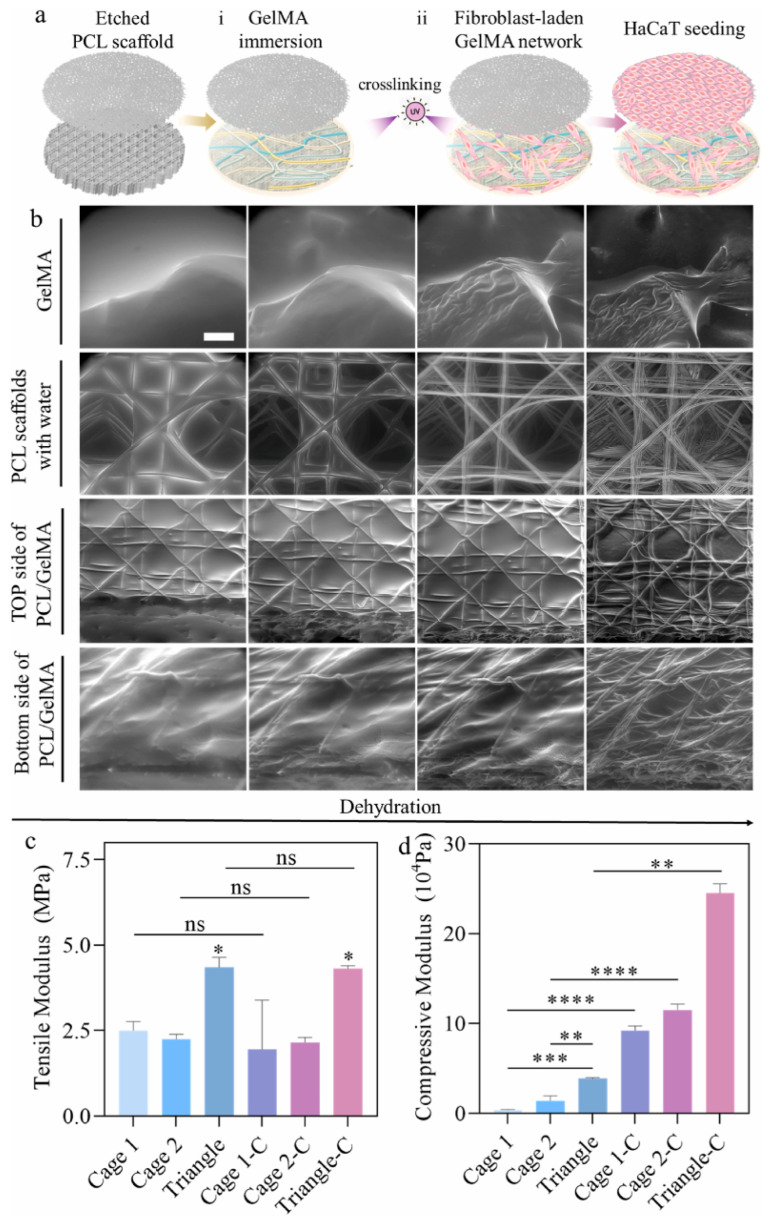
MEW–GelMA hybrid scaffold for vascularized skin equivalents. (**a**) Schematic of the dual-layer MEW scaffold design, with random fibers for the epidermal compartment and aligned fibers filled with fibroblast-laden GelMA for the dermal layer. (**b**) Histological cross-section of the optimized skin equivalent showing epidermal stratification, dermal fibroblast distribution, and vascular network integration. Scale bar: 100 μm. (**c**,**d**) Quantitative comparison of cell distribution across three dermal designs with varying porosity. The data are shown as Mean ± SD. *p* value * ≤ 0.05, ** ≤ 0.005, *** ≤ 0.001, and **** ≤ 0.0001, ns: *p* ≥ 0.05, one-way ANOVA tests, *n* = 3. Adapted from Wu et al., *Bioact. Mater.* 2025 [52], under CC BY 4.0 license.

An architecturally distinct strategy was reported by Wu et al., who combined MEW-fabricated PCL scaffolds with fibroblast-laden GelMA hydrogel to produce pre-vascularized three-dimensional skin equivalents [52]. The scaffold comprised two spatially distinct layers: a random fiber apical region supporting keratinocyte-based epidermal reconstruction, and an aligned fiber basal region filled with fibroblast-laden GelMA to recapitulate the dermis. Among three dermal designs of varying porosity, the largest pore size achieved optimal cell distribution, penetration, and ECM deposition within one week. Subsequent incorporation of an endothelial cell-laden Matrigel layer introduced a cellular vascular structure, addressing for the first time the vascularization gap in a MEW-based skin construct. Scaffold architecture and histological analysis are shown in Figure 3.

Several critical limitations, however, deserve explicit attention. First, neither model incorporates skin appendages, including hair follicles, sebaceous glands, or sweat glands, whose presence is essential for full skin function and whose engineering remains an open problem across all scaffold platforms [53]. Second, the endothelial network described by Wu et al. is a cellular surrogate rather than a perfusable lumenized vasculature; its long-term stability under physiological flow conditions and its capacity to anastomose with host vasculature after implantation have not been demonstrated [54].

A further unresolved question concerns ECM composition: whether the neosynthesized ECM in bilayer SES–MEW constructs recapitulates the native collagen I/III compositional [55,56], over extended culture periods or in vivo, has not been quantified in either study. Finally, vascularization remains the most critical determinant of clinical applicability and engraftment success: despite strategies involving growth factor incorporation, endothelial cell pre-vascularization, and stromal vascular fraction co-culture, no construct has yet achieved the capillary density and perfusion kinetics required for reliable engraftment in full-thickness wounds [57]. The MEW–GelMA approach of Wu et al. is a meaningful step, but the fundamental challenge of achieving perfusable, anastomosable vasculature remains unresolved.

Comparing the two constructs directly, Girard et al. [50] achieved earlier epidermal compartmentalization and detectable elastin deposition by day 18 through the use of a discrete SES membrane as a DEJ surrogate but did not address vascularization. Wu et al. [52] introduced a cellular vascular network through endothelial cell-laden Matrigel, advancing construct complexity, but at the cost of a non-perfusable, non-lumenized vascular surrogate whose long-term stability has not been demonstrated. Neither study has been validated in vivo. These differences reflect a design trade-off intrinsic to current SES–MEW skin constructs: structural biomimicry and vascularization have not yet been achieved simultaneously in a single construct.

### 3.2. Vascular Tissue Engineering: Small-Diameter Grafts

The replacement of small-diameter blood vessels (inner diameter below 6 mm) represents one of the most persistent and clinically consequential unresolved problems in vascular surgery. Autologous vessels remain the gold standard but are limited by availability [58] and synthetic conduits fail below 6 mm primarily due to thrombosis and compliance mismatch [59,60]. The native arterial wall achieves its nonlinear anisotropic behavior through a trilayer hierarchy: circumferential SMCs in the media, helical collagen in the adventitia, and an intimal endothelial monolayer regulating thrombogenicity [61]. Recapitulating this structural hierarchy in a synthetic construct, while simultaneously ensuring adequate suturability, burst pressure, compliance matching, and long-term patency, constitutes the central engineering challenge of small-diameter vascular graft development.

**Figure 4:**
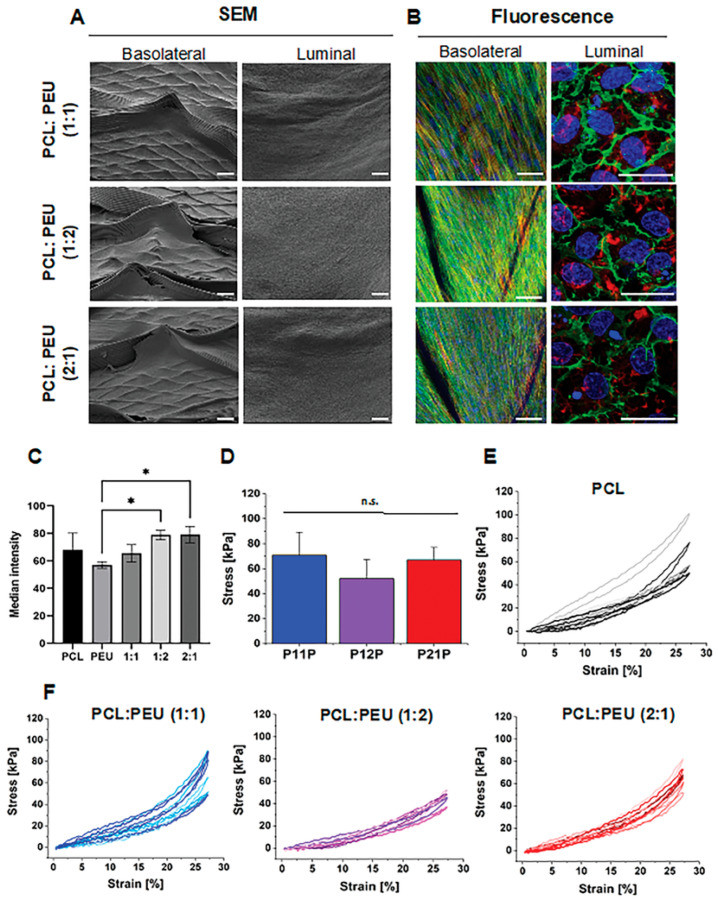
Hybrid SES–MEW bilayered vascular graft. (**A**) SEM images of the SES and MEW layers at varying polymer ratios (scale bars: 100 µm, 10 µm). (**B**) Fluorescent staining of mesenchymal stromal cells on the basolateral MEW surface (F-actin green, α-SMA red, nuclei blue) and endothelial cells on the luminal SES surface (CD31 green, Collagen Type IV red, nuclei blue) after 14 and 7 days of culture, respectively (scale bar: 25 µm). (**C**) Quantitative analysis of α-SMA intensity of MSCs seeded on co-spun constructs). The data are shown as Mean ± SD. *p* value * ≤ 0.05, one-way ANOVA tests, *n* = 3. (**D**) Mean peak stress values across co-spinning ratios. ns: *p* ≥ 0.05, one-way ANOVA tests, *n* = 5. (**E**) Stress–strain curves of the pure PCL SES membrane (*n* = 5), serving as mechanical baseline and illustrating the near-linear stiffness response in the absence of PEU. (**F**) Stress–strain curves of co-spun PCL:PEU constructs at ratios of 1:1 (blue), 1:2 (purple), and 2:1 (red) (*n* = 5–7 per group), demonstrating that PEU incorporation progressively introduces nonlinear J-shaped mechanical behavior; the 1:2 ratio achieves the most pronounced toe-region and lowest peak stress, most closely approaching the compliance profile of native vascular tissue. Adapted from Bartolf-Kopp et al., *Adv. Funct. Mater.* 2024, 34, 2311797 [62], under CC BY license.

The foundational work in this domain was reported by Jungst et al., who showed that a bilayered tubular scaffold (inner SES PCL/PU layer, outer MEW layer at controlled winding angle) directed MSCs toward near-circumferential orientation through topographic guidance alone, without soluble factors [22]. This confirms that MEW fiber geometry alone can encode cell phenotype, supporting off-the-shelf, biologic-free construct design. Bartolf-Kopp et al. demonstrated PEU content in the co-spun SES layer progressively introduces nonlinear J-shaped mechanical behavior: comparison of the pure PCL baseline (Figure 4E) with the co-spun PCL:PEU constructs at ratios of 1:1, 1:2, and 2:1 (Figure 4F) shows a progressive shift from near-linear stiffness toward the toe-region and exponential strain-stiffening characteristic of native vascular tissue, most pronounced in the 1:2 formulation [62]. In the same construct, the luminal SES surface supported confluent endothelium while the basolateral MEW surface directed MSC orientation (Figure 4B).

A mechanistically complementary approach was reported by Federici et al., who developed a three-component graft: an MEW PCL framework replicating the tunica media, a lyophilized fibrinogen ECM infused within MEW pores, and an outer electrospun PLCL adventitial layer [63]. The three-component construct closely matched native saphenous vein compliance in the 0–150 mmHg physiological range; burst pressure, while slightly lower than pure electrospun samples, remained well above the clinical threshold. HUVEC seeding confirmed the functional advantage of the hybrid intimal substrate over MEW alone, with enhanced endothelial adhesion and von Willebrand factor expression. Upon implantation as an abdominal aorta substitute in a rat model, the graft demonstrated acceptable blood interaction with reduced platelet and erythrocyte infiltration, making it one of the few SES–MEW hybrid vascular constructs to have undergone in vivo assessment.

**Figure 5:**
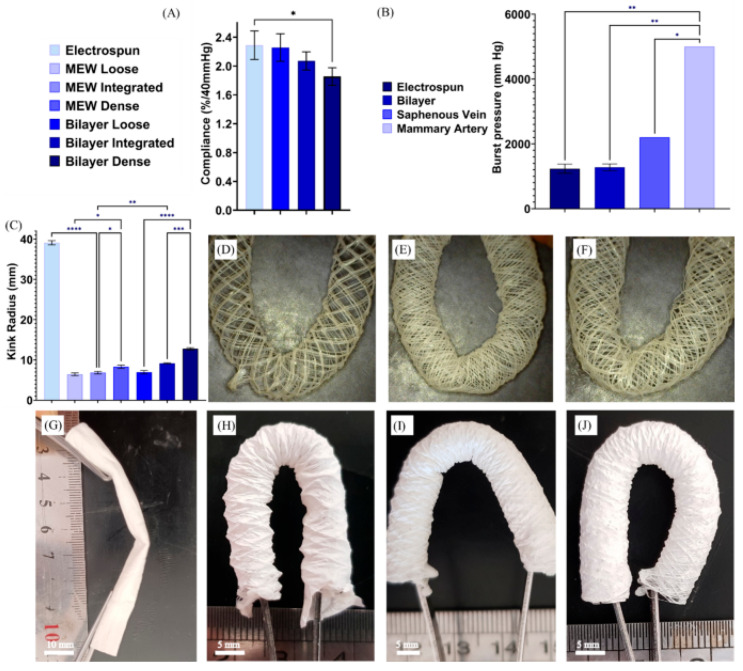
Compliance of scaffolds in the pressure range 80–120 mmHg (**A**), Burst pressure assessment (**B**), Comparing kink radius of different MEW, Bilayer, and co-electrospun scaffolds (**C**). Kink radius essay of loose (**D**), dense (**E**), and integrated (**F**) MEW stents. Kink radius assessment of co-electrospun (**G**), bilayer loose (**H**), dense (**I**), and integrated scaffold (**J**). The results are presented in average  ±  standard error, *n* = 3. (* indicates significant difference * *p* < 0.05, ** *p* < 0.01, *** *p* < 0.001, and **** *p* < 0.0001). Adapted from Shahverdi et al. [64], *Sci. Rep.* 2025, 15, 24894, under CC BY 4.0 license.

An inversion of the layer configuration was reported by Shaygani et al., who placed a MEW-fabricated TPU stent as the inner structural layer and deposited a co-electrospun heparinized PCL/PU/gelatin membrane as the outer layer, targeting the kink resistance limitation of conventional grafts at arterial bends [64] (Figure 5). The resulting bilayer construct achieved a maximum tensile strength of 8.07 ± 0.37 MPa and a burst pressure of 2.01 ± 0.1 bar, and MTT assays confirmed progressive cell proliferation over seven days.

A critical limitation shared by all SES–MEW vascular constructs reported to date is insufficient suture retention force. The Shaygani et al. construct achieved a suture retention force of only 0.37 ± 0.03 N, well below the clinical benchmarks of 1.72 N (internal mammary artery) and 1.92 N (saphenous vein) per ANSI/AAMI ISO 7198:2016 [64,65,66]. Suture retention strength is a mandatory regulatory benchmark: the stress state during suturing differs fundamentally from the in-service mechanical environment, making tensile strength and burst pressure necessary but not sufficient for surgical implantability [45].

Additional unresolved issues further complicate the translational outlook. First, none of the SES–MEW hybrid constructs reported to date has undergone long-term in vivo evaluation in a hemodynamically relevant large animal model: the Federici et al. rat model [63] does not address remodeling dynamics, thrombogenicity, or patency over clinically relevant timescales. Second, endothelial stability under physiological shear has not been assessed, as all in vitro studies use static conditions [67,68]. Third, the polymer libraries used across all reported systems (PCL, PU, PLCL, TPU) have substantially different degradation kinetics in vivo: PCL degrades over two to three years, PLCL over six to twelve months, depending on copolymer composition, while TPU-based materials are largely biostable. This inter-layer degradation mismatch creates a window of mechanical vulnerability that remains uncharacterized [69]. In summary, the SES–MEW hybrid approach for small-diameter vascular grafts remains substantially short of the biological and regulatory evidence base required for clinical translation. Bartolf-Kopp et al. [62] achieved the closest recapitulation of native J-shaped vascular mechanics through compositional co-spinning but did not report suture retention values. Shaygani et al. [64] measured suture retention explicitly, recording 0.37 ± 0.03 N against clinical benchmarks of 1.72–1.92 N [65], a gap of nearly one order of magnitude. Federici et al. [63] is the only construct to have undergone in vivo implantation, but in a rat abdominal aorta model at low hemodynamic demand. No single construct satisfies simultaneously the mechanical, biological, and surgical implantability requirements for small-diameter vascular grafts.

### 3.3. Bone Tissue Engineering

Bone represents the most structurally and biologically demanding target for hybrid scaffold design within the SES–MEW framework. Critical-size bone defects cannot heal spontaneously and autologous grafts are limited by donor site morbidity [70]. Bone is a hierarchical composite spanning nanoscale mineralized fibrils to macroscale cortical architecture, a complexity that no single fabrication technology can replicate in its entirety [71]. This is precisely the design space that SES–MEW hybrid strategies address, as outlined in Section 2.

The SES–MEW hybrid approach for bone tissue engineering has followed two complementary trajectories: one targeting bioactivity through dual payload incorporation, the other pursuing structural hierarchy through multi-technology integration. The most thoroughly characterized bioactive construct is the scaffold reported by Lai et al. [72], comprising a hierarchical nanofiber-microgrid architecture co-loaded with hydroxyapatite and roxithromycin. Hydroxyapatite promoted osteogenic differentiation while roxithromycin provided antibacterial activity in vitro.

**Figure 6:**
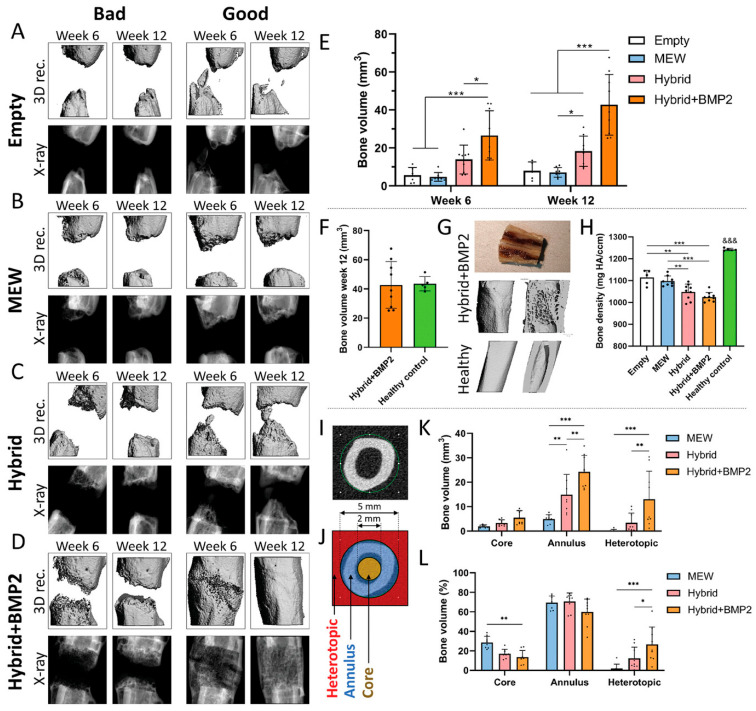
MEW–FDM hybrid scaffold with CaP nano-needle coating for critical-size bone defect repair: Analysis of bone healing using µCT imaging. Examples of good and poor healing are provided at 6 and 12 weeks for (**A**) empty, (**B**) MEW, (**C**) hybrid, and (**D**) hybrid+BMP2 groups. (**E**) Bone volume within the 5 mm defect at week 6 and 12, showing enhanced healing, in particular with the hybrid and hybrid+BMP2 groups. (**F**) Bone volume within the hybrid+BMP2 group at week 12 compared to healthy leg controls. (**G**) Macroscopic photograph of the explanted hybrid+BMP2 construct at 12 weeks (top) and comparative µCT cross-sections of the hybrid+BMP2 group versus a healthy femur control (bottom), illustrating partial restoration of cortical and trabecular architecture. (**H**) Bone density at week 12 compared to healthy leg controls. * *p* ≤ 0.05, ** *p* ≤ 0.01, *** *p* ≤ 0.001 among implant groups; &&& *p* ≤ 0.001 versus Healthy control. (**I**) µCT scan at the healthy femur midsection, which is approximately 5 mm in diameter. (**J**) Definition of heterotopic, annulus, and core regions based on scans of healthy rats. (**K**) Total bone volume and (**L**) percentage bone volume within the core, annulus, and heterotopic regions. Data presented as mean ± SD, *n* = 5–6 (empty group), *n* = 8–9 (MEW, hybrid, hybrid+BMP2 groups), *n* = 5 (Healthy control group), *p*-values are calculated using two-way ANOVA with Tukey’s multiple comparisons post-test for bone volume analyses, and one-way ANOVA with Tukey’s multiple comparisons post-test for bone density analysis at week 12. * *p* ≤ 0.05, ** *p* ≤ 0.01, *** *p* ≤ 0.001. Adapted from Eichholz et al., *Adv. Healthc. Mater.* 2024, 13, e2302057 [43], under CC BY 4.0 license.

The structural trajectory was pursued by Eichholz et al., who reported a tri-component construct combining MEW, fused deposition modeling, and a biomimetic calcium phosphate nano-needle coating [43]. The scaffold comprised an outer fused deposition modeling PCL shell preventing soft tissue collapse, an inner MEW core for osteogenic guidance, and a nanohydroxyapatite coating for osteoconductivity. The construct was evaluated in a rat critical-size femoral defect model against MEW-only and fused deposition modeling-only controls, as shown in Figure 6. The latter is organized in three blocks: qualitative µCT reconstructions by group and timepoint (A–D), quantitative bone volume and density outcomes (E, F, H), and spatial subregional analysis (I–L); panel G provides a morphological reference comparison. µCT reconstructions at 6 and 12 weeks (Figure 6A–D) show qualitatively superior healing in hybrid and hybrid+BMP-2 groups relative to MEW-only and empty controls. Quantitatively, bone volume within the defect was significantly higher in hybrid groups at both timepoints (Figure 6E), with hybrid+BMP-2 approaching values comparable to healthy contralateral controls at 12 weeks (Figure 6F). Morphological comparison of cross-sectional µCT images confirms partial restoration of cortical and trabecular architecture in the hybrid+BMP-2 group relative to a healthy femur reference (Figure 6G). Regional analysis (Figure 6J–L) revealed preferential core bone fill in the hybrid+BMP-2 group, mechanistically linked to pore geometry preservation by the FDM outer shell, which prevented soft tissue compression and enabled vascular infiltration beyond the ~200 µm diffusion limit. Bone density at 12 weeks remained below healthy controls across all groups (Figure 6H), confirming that structural consolidation precedes full mineral maturation within the observation window.

Golafshan et al. addressed periodontal ligament-to-bone interface regeneration using graded MEW scaffolds with magnesium phosphate/PCL fibers, recapitulating the functional gradient between mineralized and non-mineralized periodontium compartments in co-culture [73]. This work demonstrates that MEW alone can encode spatial biological identity through compositional gradients. No SES–MEW hybrid has been applied to this target; the Golafshan et al. construct illustrates a principle that would gain power with an SES layer enabling spatially defined bioactive delivery.

Three critical limitations remain. The first concerns vascularization: osteogenesis depends on vascular infiltration, generally requiring pore sizes above 300 µm [74,75,76]. Neither pore geometry nor degradation rate has been systematically optimized across SES–MEW hybrid constructs, and the temporal mismatch between PCL degradation kinetics and bone remodeling dynamics represents a well-documented but unresolved concern [26]. The second concerns mechanical competence in load-bearing sites. The elastic modulus of cortical bone ranges from approximately 15 to 25 GPa, depending on anatomical site and loading direction [77,78]. Hybrid construct moduli remain substantially below this range; cancellous bone equivalence may be achievable, but long-term integrity under cyclic loading has not been demonstrated. The third concerns infection management. The Lai et al. system demonstrates dual payload feasibility, but in vivo performance against biofilm-forming staphylococcal strains has not been characterized in an established infection model [79,80]. Comparing the two principal constructs, Eichholz et al. [43] provides the most complete translational dataset in the SES–MEW bone literature, with in vivo µCT quantification of bone volume and vascular infiltration in a mechanically loaded defect model, but does not address infection. Lai et al. [72] is the only construct to incorporate dual antibacterial and osteogenic payload, addressing a clinically relevant co-challenge, but lacks any in vivo validation. These two studies therefore address complementary gaps rather than competing solutions, and their integration into a single construct design represents a logical next step.

### 3.4. Soft Connective Tissues: Heart Valve Leaflets, Pericardium, and Cartilage

Among the soft connective tissues targeted by MEW-based strategies, cardiac valves and pericardium occupy a uniquely demanding position because they must simultaneously satisfy multiple and partly conflicting mechanical requirements: high compliance at low strains for leaflet coaptation, strain stiffening at higher strains to resist peak systolic pressure, and directional anisotropy enabling the energy-efficient cyclic opening and closing cycle [81,82]. The native aortic valve leaflet achieves this through a trilayer architecture whose J-shaped stress–strain response reflects sequential elastin and collagen recruitment [61,83]. Recapitulating this mechanical signature remains unresolved, with failure modes computationally linked to mechanical deviations [84,85].

**Figure 7:**
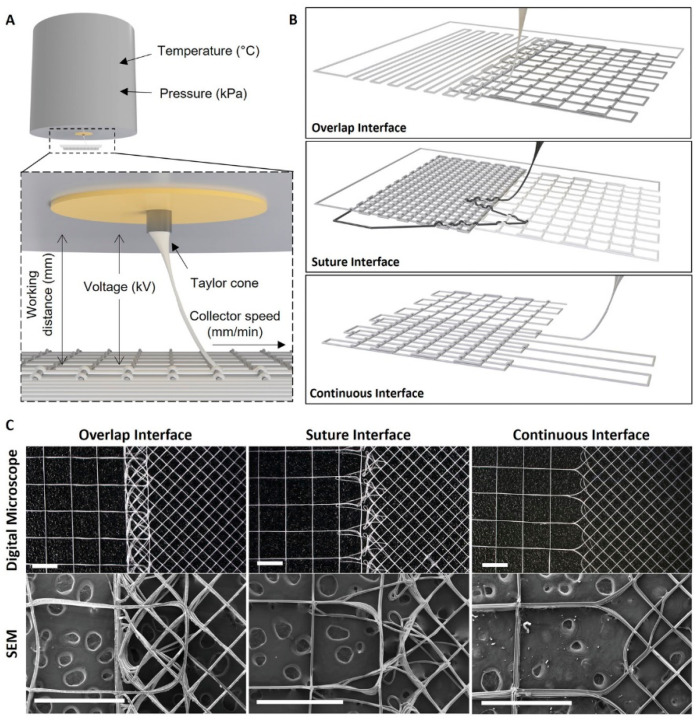
Bioinspired MEW scaffold for aortic heart valve interfaces: types of MEW interfacing methods investigated. (**A**) Schematic of the MEW system and its process parameters. (**B**) Schematic of overlap, suture, and continuous MEW interfacing methods. (**C**) Digital microscope (**top**) and SEM (**bottom**) images of MEW PCL scaffolds with 1 mm pore square patterns and 0.5 mm pore diamond patterns interfaced with the overlap, suture, and continuous methods. Scale bars = 1 mm. Adapted from Vernon et al., *Adv. Healthc. Mater.* 2022, 11, e2201028 [87], under CC BY-NC 4.0 license.

Given the mechanical programmability of MEW described in Section 2, it has become the primary fabrication platform for this challenge. The foundational demonstration of this principle in heart valve tissue engineering was provided by Saidy et al., who showed that MEW PCL scaffolds with serpentine fiber architectures recapitulate aortic valve leaflet mechanics and support collagen and elastin deposition by hVSMCs [86]. Vernon et al. used multimodal collagen mapping to design MEW scaffolds with continuous interfaces and gradient porosities, achieving yield strain, hysteresis, and stress relaxation comparable to porcine aortic valve tissue [87] (Figure 7).

The most conceptually advanced contribution in this domain was reported by Mirani et al., who coupled finite element analysis (FEA), a design of experiments (DoE) approach, and MEW fabrication into an iterative framework generating PCL scaffolds with sinusoidal fibers and prescribed biaxial properties across 65 conditions [88]. Unit cell finite element (FE) models were optimized against biaxial data from adult aortic valve, pediatric pulmonary valve, and pericardium. This framework is particularly relevant for pediatric valve replacement, where growth potential is a critical unmet need. MEW scaffolds embedded in fibrin hydrogel laden with human umbilical cord perivascular cells and cultured for 20 days yielded constructs matching native aortic and pulmonary valve stress–strain behavior within 10 ± 3% and 6 ± 2% deviation, respectively, with 95 ± 2% cell viability and progressive fibrin replacement by glycosaminoglycans and collagen. The resulting tissue sheets were then sutured into trileaflet valves, demonstrating functional performance under pulmonary pressure conditions in a pulse duplicator bioreactor.

A complementary approach was pursued by Xu et al., who combined MEW-fabricated anisotropic PCL scaffolds with a GelMA/chondroitin sulfate methacrylate bioactive hydrogel to engineer a composite heart valve construct [89]. MEW-PCL provided structural anisotropy while the hydrogel supplied microporosity for VIC infiltration; ChsMA incorporation improved hemocompatibility and endothelialization in vitro. In vivo implantation in Sprague-Dawley rats demonstrated that the PCL-GelMA/ChsMA construct suppressed immune reactions and calcification more effectively than PCL-only scaffolds.

MEW, alone or combined with hydrogel phases, is currently the most capable platform for soft connective tissue constructs requiring prescribed, region-specific mechanical behavior. However, several critical gaps remain. Most critically, no construct has been tested under aortic hemodynamic loading: Mirani et al. used a pulmonary pressure pulse duplicator, and the Xu et al. rat model provides no hemodynamic challenge [88,89]. Integration of an SES component for ECM-mimetic nanotopography and sustained growth factor delivery has not been systematically investigated in any valve construct [20].

Articular cartilage shares two design requirements with cardiac valve tissue: nonlinear anisotropic mechanical recapitulation and spatially defined interfaces between mechanically distinct compartments [90]. Visser et al. established that MEW-reinforced GelMA hydrogels achieve compressive properties substantially above unreinforced controls while supporting chondrocyte viability [91]. De Ruijter et al. extended this to patient-specific geometries, developing a zonally arranged MEW-PCL/GelMA osteochondral implant shaped from the medial femoral condyle with neocartilage formation after 28 days [92]. An electrospun membrane at the cartilage–bone interface has been shown to replicate the native tidemark by preventing cell migration and establishing a mineral concentration gradient comparable to native osteochondral tissue [93]. The combination of MEW structural reinforcement at the cartilage scale with SES nanofibrous membrane integration at the tidemark interface represents a design logic that addresses both mechanical and biological gradient requirements simultaneously, but has not yet been realized in a single integrated construct.

### 3.5. Skeletal Muscle

Skeletal muscle is among the most architecturally demanding targets in tissue engineering. The myofiber, the functional unit of skeletal muscle, is a polarized anisotropic structure whose MHC expression, sarcomere assembly, and myotube formation are regulated by ECM topographic and mechanical cues [94]. Native muscle ECM anisotropy drives myoblast elongation and differentiation through integrin-mediated mechanotransduction [95]. Scaffolds must replicate this guidance at the relevant length scale, including fiber diameters of 10–100 µm with inter-fiber spacings supporting both myogenic fusion and vascular access [96]. Volumetric muscle loss (VML) represents the paradigmatic clinical context for these constructs: satellite cell-mediated endogenous regeneration fails because the native biophysical and biochemical environment has been obliterated, and autologous tissue transfer achieves incomplete functional recovery with significant donor site morbidity [97].

**Figure 8:**
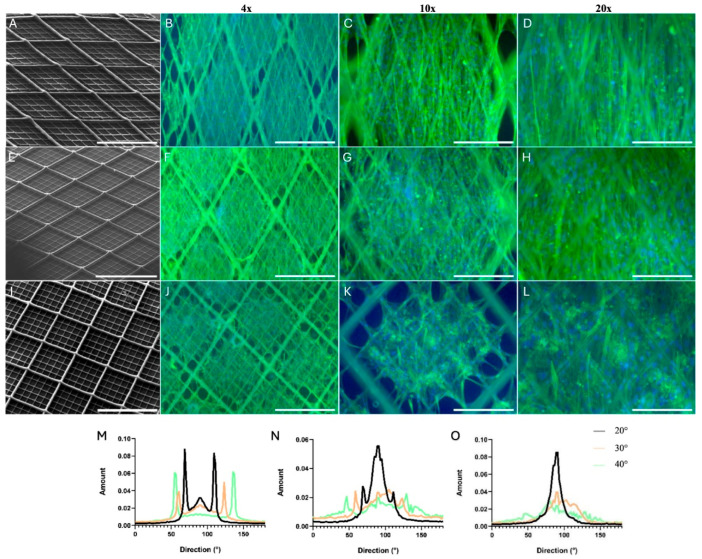
MEW PCL scaffolds for human skeletal muscle cell alignment: Skbm2 cell alignment on MEW scaffolds (**A**–**D**) 20°, (**E**–**H**) 30°, and (**I**–**L**) 40°. Scale bars and corresponding alignment histograms for 4× mag: 1 mm (**M**), 10× mag: 400 µm (**N**), 20× mag: 200 µm (**O**). SEM scale bars (**A**,**E**,**I**) 2 mm. (**M**) Resultant histogram after analysis at (**M**) 4× (**N**) 10×, and (**O**) 20× magnification. For all histograms, black, orange, and green represent the 20°, 30°, and 40° scaffolds, respectively. Adapted from Snow et al., *Biofabrication* 2025, 17, 035013 [98], under CC BY 4.0 license.

Within this context, MEW has recently emerged as a high-resolution platform well suited to the directional guidance requirements of skeletal muscle tissue engineering. Snow et al. provided the most systematic characterization to date of the relationship between MEW fiber orientation and human skeletal muscle cell behavior, culturing primary myoblasts and dermal fibroblasts on PCL scaffolds fabricated at three fiber angles (20°, 30°, 40°) [98]. Uniaxial strain testing defined an optimal strain regime for transitioning from static to cyclic culture. Cell alignment was highest on 20° scaffolds, confirming inter-fiber pore geometry as the primary determinant of topographic guidance (Figure 8). Desmin staining confirmed myogenic identity across all designs.

Fiber diameter and inter-fiber spacing are particularly critical: MHC expression is significantly higher on parallel fibers and at 100 µm versus 200 µm inter-fiber spacing, confirming that topographic cue scale must match the fusing cell population [99,100,101]. MEW extends this framework with the geometric precision described in Section 2 [102].

Clinically relevant VML defects cannot be addressed with two-dimensional fiber mats or thin scaffolds; volumetrically significant constructs require integrated vascularization strategies that have not yet been realized in any MEW-only system [103]. Aligned electrospun nanofibers promote endothelial cell elongation, vascular network formation, and anastomosis with host vasculature in VML models. Aligned collagen scaffolds improved microvessel density relative to non-aligned controls, with myoblast–endothelial co-culture further enhancing vessel density [104]. VEGF-expressing HUVEC/ASC co-culture on parallel PCL/gelatin nanofibers upregulated MyHC2 expression, with SEM confirming direct myoblast–endothelial contact [105].

The SES–MEW hybrid logic for skeletal muscle inverts the usual functional assignment: MEW directs myoblast alignment and defines the myotube mechanical framework, while SES provides the nanofibrous interstice within which endothelial cells organize into capillary networks and deliver angiogenic factors in a spatially localized manner [106]. Yeo et al. combined HUVEC-laden electrospun alginate fibers with 3D-printed PCL/collagen supports, showing that aligned SES directed HUVEC elongation into capillary networks while enhancing myogenic marker expression in co-cultured C2C12 myoblasts [107]. When upscaled to a 50 mm diameter construct, the co-culture approach produced significantly enhanced vascular density in vivo, with vessel length density increasing from 3.68 to 5.03 mm/mm^2^ [106]. This result confirms that interstitial vascularization requires a spatially organized angiogenic cellular component, a function that SES is uniquely positioned to provide at the nanoscale. Comparing the two vascularization approaches, Yeo et al. [107] achieved myoblast–endothelial co-culture on aligned SES/3D-printed PCL constructs with confirmed myogenic marker upregulation, but at a 2D construct scale insufficient for volumetric muscle loss applications. Borisov et al. [106] demonstrated upscaled constructs (50 mm diameter) with significantly enhanced in vivo vascular density (vessel length density 3.68 to 5.03 mm/mm^2^), but used non-MEW 3D-printed PCL supports without an SES component. Neither study constitutes an integrated SES–MEW hybrid for skeletal muscle, confirming that this combination, despite strong mechanistic rationale, remains unrealized experimentally.

Despite this convergence of evidence, an integrated SES–MEW hybrid scaffold for skeletal muscle has not yet been reported. The fabrication and biological challenges involved are technically tractable, as discussed in Section 5.1, and the existing evidence base supports prioritizing the SES–MEW combination in skeletal muscle as the next experimental step in this domain.

## 4. Critical Cross-Tissue Analysis

The five tissue domains examined in the preceding sections define a landscape in which SES–MEW hybrid strategies have been applied with different degrees of biological validation, architectural sophistication, and translational readiness. A comparative rather than sequential analysis reveals cross-domain patterns that would remain invisible in a tissue-specific analysis: recurring structural logics, shared failure modes, and a set of principles that appear to be domain-independent.

The most consistent finding across all five domains is that MEW and SES contribute non-redundant and genuinely complementary functions to every hybrid construct in which they have been combined. As established in Section 2, MEW provides the architectural backbone and SES the bioactive nanoscale interface; this complementarity is structural and non-redundant across all five tissue domains reviewed [108,109,110]. The hybrid is therefore not a compromise but a genuine design space expansion.

A second pattern that emerges across domains is the asymmetric validation profile of SES–MEW constructs: across all five tissue targets, the most advanced demonstrations are in vitro, the in vivo data are sparse and largely confined to small rodent models, and large animal or human-scale studies are absent. The most advanced in vivo data are summarized in Table 2. None of these constitutes hemodynamically loaded in vivo validation in a large animal model at a clinically relevant scale, and in the two soft tissue domains with the most developed in vitro data, skin and skeletal muscle, no in vivo study of any SES–MEW hybrid construct has been reported. This asymmetry reflects a well-recognized limitation of tissue engineering research broadly, where cost, ethical constraints, and scale mismatches between small animal models and human constructs collectively impede translation [111,112], compounded by the scale-up challenges discussed in Section 5.3.

A third pattern, less discussed in the tissue-specific literature but critical for translational assessment, concerns the temporal mismatch between scaffold degradation and host tissue remodeling. The near-universal use of PCL across all reviewed constructs, and its slow in vivo degradation kinetics [113,114,115,116], creates tissue-specific temporal mismatches discussed in detail in Section 5. This degradation profile is reasonably matched to bone remodeling in large defects, moderately matched to the vascular wall remodeling window, and poorly matched to the rapid ECM deposition kinetics of skin, which occur over weeks, and is particularly mismatched with the vascularization requirements of skeletal muscle, where capillary ingrowth must precede myofiber survival. Although PLA, PLCL, and polyurethane derivatives offer shorter degradation profiles [117,118], systematic optimization of degradation kinetics to match the biological remodeling timeline of a specific tissue target has not been reported for any SES–MEW hybrid construct. This is a gap that is technically addressable through copolymer selection or the design of enzymatically degradable MEW inks [119,120]. It is worth noting that dual-scale scaffold strategies are not exclusive to the SES–MEW combination. Hybrid constructs integrating extrusion-based 3D printing with conventional solution electrospinning have been explored across several of the same tissue targets reviewed here, with 3D-printed frameworks providing macroscale mechanical support and defined pore geometry while electrospun membranes supply nanoscale ECM-mimetic cues [121,122]. This configuration replicates the same dual-scale functional logic as SES–MEW hybrids. The critical distinction is resolution: MEW achieves fiber diameters and pore geometries one to two orders of magnitude smaller than extrusion-based FDM or robocasting, enabling architectural control at the cell-relevant 10–100 µm scale that conventional 3D printing cannot replicate [122]. SES–MEW hybrids therefore represent a higher-resolution implementation of the same dual-scale design principle, with correspondingly greater biological fidelity at the microscale but greater fabrication complexity, a narrower polymer library, and lower throughput relative to 3D printing–electrospinning systems [121].

The comparative overview of tissue-specific SES–MEW advantages, principal open gaps, and translational readiness across the five domains is summarized in Table 2.

**Table 2:** Cross-tissue comparative overview of hybrid SES–MEW scaffold strategies. For each tissue domain, the principal SES contribution, the principal MEW contribution, the key advantage of the hybrid combination, the most critical unresolved gap, an assessment of translational readiness level (TRL, adapted from the European Commission nine-point framework), and available in vivo validation are indicated.

Tissue	Principal SES Contribution	Principal MEW Contribution	Key Hybrid Advantage	Critical Open Gap	In Vivo Validation	TRL
Skin	DEJ biomimicry; keratinocyte barrier; ECM permeability control	Open-pore dermal scaffold; fibroblast organization	Full-thickness skin equivalent in 18 days; elastin deposition from day 18 [50]	No skin appendages [52]; no perfusable vasculature [54,57]; no in vivo data	None for SES–MEW construct	3
Vasculature (SDVG)	Confluent luminal endothelium; adventitial mechanical reinforcement; ECM nanotopography	J-shaped stress-strain; circumferential MSC orientation; kink resistance	Native J-curve recapitulation; cell organization without soluble factors [62]	Suture retention below native artery [45,65]; no large animal model; compliance mismatch uncharacterized under in vivo conditions [60]; no shear-conditioned endothelial data	Rat abdominal aorta [63]	3–4
Bone	Dual payload (HAp + ROX); osteoconductive nanofiber surface; drug delivery	Microgrid mechanical backbone; FDM shell for soft tissue exclusion; pore geometry preservation in vivo	Dual osteogenic-antibacterial function [72]; vascularization superior to MEW alone [43]	In vivo antibacterial validation absent; insufficient pore geometry optimization for vascularization [76]; compressive modulus below cortical bone [77,78]; PCL degradation mismatched to bone remodeling timeline [115]	Rat femoral critical-size defect [43]	4
Heart valve/pericardium	ECM-mimetic nanotopography for VIC/VSMC; potential growth factor delivery	Prescribed biaxial nonlinear anisotropic mechanics [86,87,88]; serpentine and sinusoidal fibers from bioinspired and FEA-DOE design framework	Biaxial mechanical target matching within 10% deviation; trileaflet valve under pulmonary pressure	No fatigue data; no aortic-pressure in vivo test; SES not yet integrated in valve constructs	Subcutaneous rat (non-hemodynamic) [96]	3
Skeletal muscle	Interstitial vascularization via endothelial cell guidance [106,107]; VEGF delivery; nano-ECM for myoblast adhesion	Directional fiber orientation guiding myoblast alignment and MHC expression [98]	Myoblast alignment and myogenic differentiation dependent on fiber aspect ratio; angiogenic co-culture synergy [107]	No integrated SES–MEW hybrid reported [98]; vascularization unresolved at the construct scale [103,104]	None for SES–MEW construct	2

Reading Table 2 across the TRL column reveals a clear asymmetry: bone leads at TRL 4, supported by the only hybrid construct tested in a mechanically loaded in vivo defect model [43]; vasculature follows at TRL 3–4, with one rat implantation study at low hemodynamic demand [63]; and skeletal muscle occupies TRL 2, as no SES–MEW hybrid construct for this target has yet been fabricated [107]. Reading the table along the Critical open gap column reveals that vascularization is the recurring unresolved problem in every soft tissue domain, while mechanical competence is the limiting challenge in hard tissue, and mechanical fatigue under cyclic loading is the limiting challenge for valve leaflets.

This cross-tissue analysis also reveals a structural inversion in the field’s current development trajectory. The mechanically simpler tissue applications, skin and skeletal muscle, are biologically more demanding in terms of vascularization, cellular complexity, and appendage formation; the applications that are mechanically most demanding, bone cortex and cardiac valve, are simpler biologically in terms of cell type diversity and vascularization requirements, at least at the early stages of in vitro maturation. SES–MEW hybrid development has disproportionately advanced in the mechanically demanding domains because mechanical characterization provides quantifiable targets that can be addressed by scaffold design iteration; vascularization is a systems-level problem requiring co-culture, growth factor delivery, and in vivo assessment, not amenable to incremental in vitro iteration [123,124]. The practical consequence is that the most clinically urgent applications, skin grafts for large burns and skeletal muscle constructs for VML, are also the least translationally mature in the SES–MEW literature.

Finally, the cross-tissue analysis supports one overarching mechanistic conclusion: the SES component is not interchangeable across applications but must be tissue-specifically designed. In skin, its function is as a DEJ surrogate and cell compartment barrier; in vasculature, as a luminal thromboresistant interface and compliance-modifying outer adventitia layer; in bone, as a dual-payload delivery vehicle for osteogenesis and infection control; in heart valves, as a potential VIC-supporting interface whose integration with the MEW mechanical backbone has not yet been realized; in skeletal muscle, as a pro-vascular nanofiber template. These are fundamentally different design requirements across domains, and they preclude any one SES formulation or architecture from serving all tissue targets simultaneously. This tissue-specificity of the SES component design is the central insight generated by comparing the five domains side by side, and it implies that the translational development of SES–MEW hybrid constructs must proceed as five distinct but interrelated programs rather than as a single generalized platform [39,125,126].

## 5. Translational Barriers

The gap between the current state of the literature and the first clinical use of any SES–MEW hybrid construct remains wide. This section examines the structural barriers to clinical translation that are not tissue-specific but systemic in nature: process standardization and reproducibility, regulatory classification and biocompatibility requirements, scale-up manufacturing, and the emerging role of machine learning in closing the loop between fabrication parameters and output quality. Process standardization and machine learning are treated together, the latter being most relevant as a solution to the former.

### 5.1. Process Standardization, Reproducibility, and the Role of Machine Learning

As described in Section 2, MEW is a fundamentally multiparametric process [38]. The MEW jet is electrohydrodynamically stabilized by a balance between electrostatic and viscous forces that is highly sensitive to any of these variables, such that a change in one requires re-optimization of several others [127,128]. The transition of MEW from academic laboratories to industrial manufacturing has been hampered by slow experimentation cycles, low printing throughput, poor output reproducibility, and a high degree of user-dependent operation arising from the nonlinear and multiparametric nature of the process [129,130]. Solution electrospinning faces an identical challenge; in the SES literature, systematic characterization of process–structure–property relationships using high-throughput design of experiments (DoE) approaches has improved reproducibility, yet no universally agreed reporting standard for SES scaffolds exists [131]. For SES–MEW hybrids, the electrostatic interference described in Section 2 must also be co-optimized [132,133]. Establishing inter-laboratory reproducibility standards for SES–MEW hybrid constructs is therefore a prerequisite for any regulatory submission.

Mieszczanek et al. demonstrated a technically important step toward resolving the MEW reproducibility problem by implementing a closed-loop neural network control system using real-time computer vision of the MEW jet to dynamically adjust printing parameters [134]. This approach reduced optimization time from days to hours and improved dimensional reproducibility compared to open-loop operation [134,135]. The approach provides a technology-agnostic template extendable, in principle, to simultaneous SES–MEW systems if analogous process signatures for the SES component were identified. Recent advances in high-speed imaging and machine learning-based prediction of electrospinning jet profiles [136], and in physics-informed Bayesian frameworks for modeling electrohydrodynamic jet dynamics [137], suggest that this extension is technically feasible. The use of machine learning not only improves reproducibility but also generates the structured, quantitative process data that regulatory agencies require to assess the manufacturing consistency of a medical device, directly linking the reproducibility problem to the regulatory challenges discussed in the following section.

### 5.2. Regulatory Classification and Biocompatibility

SES–MEW hybrid constructs for implantable tissue engineering applications fall, in the European regulatory framework, under Medical Device Regulation (EU) 2017/745 as Class III devices, given their long-term implantable nature [46,138]. The specific requirement under MDR Annex I that manufacturers minimize the risk posed by contaminants and residues to patients is directly applicable to the SES component of any SES–MEW hybrid, because SES relies on organic solvents whose complete removal must be documented per ISO 10993-17:2023 and ISO 10993-18:2020 [139,140]. Residual solvents such as HFIP and DMF carry established toxicity profiles requiring full extractables and leachables quantification prior to any clinical authorization [41]. Although MEW is solvent-free and generates a simpler chemical dossier, the hybrid construct inherits the full regulatory burden of its SES component [141].

The classification of cell-seeded SES–MEW constructs further complicates the regulatory pathway, because such constructs fall under the ATMP framework, requiring EMA authorization and GMP-compliant manufacturing [142]. This regulatory ambiguity is particularly acute for SES–MEW hybrid systems because the constructs most advanced in terms of biological validation are precisely those that would fall under the more demanding ATMP framework, creating an inverse relationship between scientific maturity and regulatory accessibility. To date, no SES–MEW hybrid construct has entered clinical trials in any jurisdiction. The most advanced constructs reviewed here, the MEW–FDM bone scaffold [43] and the three-component vascular graft [62], have been evaluated exclusively in small rodent models at TRL 3–4, substantially below the TRL 6–7 threshold required for first-in-human submissions. The absence of clinical trial data is therefore not a limitation of this review but a direct reflection of the current state of the field. The full regulatory pathway from the current TRL to clinical authorization involves large animal validation, GMP-compatible manufacturing scale-up, full extractables and leachables characterization for the SES component, and, for cell-seeded constructs, EMA authorization under the ATMP framework. On current trajectories, first-in-human studies for bone and vascular applications represent a realistic five-to-ten-year horizon, conditional on resolution of the manufacturing reproducibility and large animal validation gaps identified in this review. Regulatory frameworks in the US, Japan, and China impose analogous but structurally distinct requirements, precluding a single harmonized global submission strategy [143,144].

### 5.3. Scale-Up Manufacturing

The fabrication of SES–MEW hybrid constructs at the dimensions required for clinical use poses a substantial scale-up challenge. MEW throughput is constrained by the critical translation speed requirement to avoid fiber buckling [145,146]. For constructs at clinically relevant dimensions, such as femoral defect implants, valve leaflets, or skin grafts, printing time would extend to several hours per construct, a throughput constraint directly quantified in the MEW literature [147,148]. Parallelization through multi-nozzle MEW printing has been demonstrated as a partial solution: Wunner et al. designed and validated a high-throughput MEW platform with eight simultaneously extruding heads capable of producing 1152 scaffolds in 87 h, demonstrating identical fiber architectures to single-head systems and large-scale lattice fabrication at 780 mm × 780 mm [149]. However, this approach improves batch throughput without reducing per-scaffold printing time, and electrostatic repulsion between adjacent nozzles constrains nozzle spacing and deflects fiber trajectories from programmed paths [150,151]. This interference is mechanistically analogous to the electrostatic crosstalk discussed in Section 5.1 [152]. The SES component adds a further manufacturing bottleneck: solvent removal by vacuum drying near the polymer glass transition temperature adds processing time and has been shown to alter fiber mechanical properties and the release kinetics of incorporated bioactive agents [41].

## 6. Future Perspectives

Building on the cross-tissue analysis in Section 4, the translational landscape reviewed here is one of considerable scientific momentum but incomplete biological validation, and the central challenge of the coming decade is to translate constructs that are demonstrably sophisticated in vitro into devices that survive biological complexity at a clinically relevant scale.

Three convergent trajectories define the most scientifically grounded near-term directions. The first and most pressing trajectory concerns vascularization. Without a pre-formed or rapidly inducible vascular network, constructs above the oxygen diffusion limit of approximately 200 µm cannot sustain cell viability after implantation [153,154,155]. The SES component is mechanistically the most suitable element for encoding vascularization: aligned SES nanofibers promote endothelial elongation and capillary formation, and enable spatially localized angiogenic factor delivery at gradients unachievable by passive diffusion from MEW substrates [156,157]. This requires SES formulations that are bioactive, mechanically compatible with MEW, and solvent-free for regulatory compliance with ISO 10993-17/18. Hydrogel electrospinning and aqueous spinning solutions represent technically concrete paths toward solvent-free SES components compatible with hybrid fabrication [158].

The second trajectory concerns material diversification of both the MEW and SES components beyond PCL and synthetic polymers. PCL degradation kinetics are poorly matched to the remodeling timescale of skin and skeletal muscle, and its semi-crystalline stiffness profile is not suited to constructs under fatigue loading, such as cardiac valves and vascular grafts [114]. Polyurethane elastomers and PLCL, already processable via MEW, would expand the mechanical and degradation design space of hybrid constructs [159,160]. MEW inks formulated from bioactive materials, such as peptide-functionalized elastomers or calcium phosphate composites, would distribute osteoinductive activity across both scaffold layers rather than concentrating it in the SES component [72]. The SES component equally benefits from material diversification beyond synthetic polymers. Silk fibroin, derived from *Bombyx mori*, represents a particularly compelling candidate: it exhibits tunable mechanical properties spanning the stiffness range of soft connective tissues, supports cell adhesion and proliferation without surface functionalization, and degrades through proteolytic pathways with a minimal inflammatory response [161]. Electrospun silk fibroin membranes have demonstrated superior biocompatibility and ECM-mimetic architecture across skin, vascular, bone, and cartilage targets [162]. Spidroin-based materials derived from spider silk further extend this material space, combining exceptional tensile strength with high cytocompatibility, as demonstrated in full-thickness skin wound models where electrospun fibroin/spidroin scaffolds achieved volume porosities above 94% with non-cytotoxic behavior [163]. Integration of silk fibroin or spidroin as the SES component in SES–MEW hybrid constructs would introduce a biologically active, solvent-compatible nanofibrous interface with inherently superior biocompatibility relative to PCL- or PU-based electrospun layers. Cryo-electrohydrodynamic jetting of aqueous silk fibroin solutions, recently demonstrated by Reizabal et al. [127], represents a technically concrete path toward solvent-free silk-based SES components compatible with hybrid fabrication.

The third and most speculative trajectory concerns simultaneous SES–MEW co-deposition, which remains at TRL 2–3 because the MEW electrostatic field disrupts the SES whipping instability on a shared collector [164,165]. A true simultaneous co-deposition system would achieve nano-micro spatial interpenetration that no sequential approach can replicate, representing the most complete realization of the hierarchical scaffold logic underlying this platform.

## 7. Conclusions

This review has examined hybrid SES–MEW scaffold strategies across five tissue domains and identified a consistent structural pattern: the two platforms occupy non-overlapping regions of a fabrication design space that map directly onto non-overlapping biological functions. MEW provides programmable microarchitecture, directional mechanical response, and topographic guidance at the 10–100 µm scale; SES provides nanotopographic cell instruction, ECM-mimetic surface chemistry, and spatially controlled bioactive delivery at the sub-micron scale. No single-platform construct reviewed here satisfies all three requirements simultaneously, and the cross-tissue analysis in Section 4 confirms that this limitation is structural rather than incidental. The SES–MEW combination is therefore not a fabrication convenience but a design necessity: the minimum configuration required to simultaneously address the mechanical, biological, and vascular demands of complex tissue targets.

The field has produced results of genuine scientific significance: recapitulation of J-shaped nonlinear anisotropic valve mechanics to within 10% biaxial deviation, dual osteogenic-antibacterial payload delivery with superior vascularization over MEW-only controls, directed vascular cell organization without exogenous soluble factors, and full-thickness skin equivalents with neosynthesized ECM in eighteen days. These are proof-of-concept validations of distinct design principles, not incremental refinements.

The unresolved challenges are equally consistent: vascularization at the volumetric scale, PCL degradation mismatch in soft tissue domains, suture retention below the native arterial threshold, absent fatigue data for valve constructs, and the absence of large animal in vivo validation for any SES–MEW hybrid. None of these is a fundamental scientific obstacle. All are tractable through material diversification beyond PCL, solvent-free aqueous and hydrogel-based SES, closed-loop machine learning process control, and co-deposition system engineering informed by electrostatic field modeling.

The translational outlook is realistic. For bone and vasculature, the gap between TRL 3–4 and the TRL 6–7 required for first-in-human submissions is a five-to-ten-year problem, conditional on large animal validation and manufacturing reproducibility. For skin and skeletal muscle, the timeline is longer, gated by the vascularization problem. For cardiac valves, the mechanical framework is the most sophisticated in the field, but hemodynamic in vivo validation remains absent. The principal bottleneck is no longer conceptual: the SES–MEW hybrid approach is scientifically sound, biologically motivated, and technically executable. What the field now requires is not a new idea but the sustained commitment to carry a proven concept through the biological, manufacturing, and regulatory complexity that separates a laboratory demonstration from a clinical device.

## Figures and Tables

**Table 1:** Comparative overview of solution electrospinning (SES), melt electrowriting (MEW), and hybrid SES–MEW constructs in the context of tissue engineering scaffold fabrication.

Parameter	SES	MEW	SES + MEW
**Fiber diameter**	100 nm–~10 µm	3–100 µm	Dual-scale: nm + µm
**Material phase**	Polymer solution	Polymer melt	Both
**Solvent required**	Yes (organic)	No	Partially (SES component)
**Process temperature**	Room temperature	65–230 °C	Both
**Applied voltage**	10–30 kV	3–10 kV	Independent per head
**Fiber deposition**	Random/aligned (whipping)	Programmed (stable jet)	Complementary
**Pore size**	Sub-micron to ~10 µm (small)	50 µm–~1 mm (tunable)	Hierarchical
**Cell infiltration**	Poor (fiber density-limited)	Excellent	Layer-dependent
**Mechanical properties**	Low stiffness, anisotropic	Tunable, programmable	Enhanced, composite
**ECM mimicry**	High (nanotopography)	Low	High (via SES component)
**Scalability**	Moderate to high (multi-nozzle compatible)	Low (single nozzle)	Limited
**Regulatory risk**	Moderate (solvent residues)	Lower	Composite (solvent residues + device classification)

## Data Availability

No new data were created or analyzed in this study. Data sharing is not applicable to this article.
